# 

**Published:** 1915-02

**Authors:** 


### ANNOUNCEMENTS.

NATIONAL MOUTH HYGIENE ASSOCIATION.—A series of illustrated lectures on Mouth Hygiene is being prepared by this association for rental service. The first lecture of the series, a talk suitable for a mixed adult audience or school pupils above the age of twelve years (designated as lecture "A") is now ready. The lecture set (manuscript and 36 slides) will be furnished to members of state dental societies and others who may be considered as competent to present the matter to the public, at a fee of one dollar per use.

For further particulars and application blanks, address the Director of Extension Lectures,

EDWIN M. KENT, D.M.D.,
222 Washington St., Brookline, Mass.

---

INDIANA STATE DENTAL ASSOCIATION.—The next annual meeting of the Indiana State Dental Association will be held on May 18, 19 and 20, 1915. A distinctive feature of this meeting will be that the program will be made up exclusively of Indiana dentists. A cordial invitation is extended to members of other dental societies to attend the meeding.

A. R. ROSS, *Secretary*, Lafayette, Ind.

---

THE OKLAHOMA STATE DENTAL SOCIETY.—The next meeting of the Society will be held in Oklahoma City, March 15, 16, 17, 18, 19, 1915. The meeting will be conducted somewhat along the same lines (the post-graduate plan) that have proven so valuable in this state the past few years. The principal lecturers will be Drs. J. H. Prothero and W. H. G. Logan of Chicago. Dr. Prothero's lectures will be confined to important phases of Prosthetic Dentistry, while Dr. Logan will give lectures on modern methods of treating Pyorrhea, and on local anesthesia. Reputable dentists from out of the state are welcome to this meeting, but are required to pay a fee of five dollars for the lectures and clinics.

C. R. LAWRENCE, *Secretary*, Enid, Okla.

---

TEXAS STATE DENTAL ASSOCIATION.—The Thirty-fifth Annual Meeting of the Texas State Dental Association will be held in Galveston, Texas, May 19, 20, 21 and 22, 1915. The special feature of this meeting will be post-graduate lectures and clinic work.

Dr. G. Walter Ditmar, of Chicago, will present modern scientific bridge work and removable partial dentures, with preparations, technique, and principles involved. Dr. B. F. Thielen will present "Plate Work"; Dr. T. G. Duckworth, "Orthodontia"; Dr. R. D. Griffis, "Nitrous Oxide Analgesia, and Anaesthesia"; Dr. Julian Smith, "Pyorrhea"; Dr. J. M. Murphy, "Local Anaesthesia."

Exhibitors are requested to attend and to write Dr. A. L. Frew, Dallas, for space. General clinics, last day. Clinicians write Dr. W. H. Nugent, Fort Worth. For any other information, write the Secretary, W. O. Talbot.

---

INTERNATIONAL JOURNAL OF ORTHODONTIA. —This is a new dental journal devoted to the subject of Orthodontia. It is edited by Dr. Martin L. Dewey, of Kansas City, the well-known writer and teacher of this specialty. It is published by the C. V. Mosby Co., of St. Louis. The first number is just out and it is a credit to the editor and the publishers as well. If there is room for a journal devoted to this subject entirely, this number promises to fill the need to the entire satisfaction of this specialty's most enthusiastic supporters. The matter is high grade and it is attractively printed on good paper, and the press work of such character as to bring out the illustrations distinctly. We cordially welcome the new journal, and trust the profession will give it hearty support. It is the first and only journal devoted exclusively to this subject in our country.

---

DENTAL BUR.—The *Dental Bur,* published by the alumni of the Chicago Dental College, has changed a quarterly publication with a new editor in the person of Dr. P. G. Puterbaugh. If he proves as entertaining a writer as his predecessor, Dr. Tuller, we shall gladly welcome him to the editorial field of dental journalism. Dr. Tuller has been compelled to give up his editorial work because of his continued bad health. He has been a most interesting and capable writer and we shall miss his witty and wholesome comments on current events.

---

ARIZONA STATE DENTAL SOCIETY.—The annual meeting of this Society was held at Phoenix, November 11th to 13th, and the following officers were elected: President, H. H. Wilson, of Phoenix; Vice-President, L. A. Hawkins, of Jerome; Secretary-Treasurer, J. L. O'Connell, of Phoenix.

---

DELAYED PUBLICATION.—Owing to a change of printers the January and February issues of the REGISTER are unusually delayed. We hope to get out in good time with future issues.

---

THE PANAMA-PACIFIC DENTAL CONGRESS is to be held at San Francisco, Cal., August 30 to September 9, 1915. The Transportation Committees are recommending the following plans and schedules of railway rates from New York, Chicago and other points to San Francisco and return.

Following the usual custom and in order that all those who desire to attend the Panama-Pacific Dental Congress at San Francisco, August 30 to September 9, 1915, may do so with the maximum of comfort and pleasure and minimum of fatigue and inconvenience, the Transportation Committees, announce that arrangements have been made for special train service. The present plan is to have three special trains from Chicago leaving as follows:

*First train*, leave Chicago on August 21st, going via Burlington Route to Kansas City and the Santa Fe to Los Angeles and Southern Pacific to San Francisco. Stop-overs will be made at Colorado Springs, Isleta Indian Village, the Grand Canyon, Redlands, Riverside, San Diego, Los Angeles, San Francisco.

*Second train*, leave Chicago on August 24th, going via Burlington Route to Denver, thence via Denver & Rio Grande to Salt Lake City and Western Pacific to San Francisco. Train two includes stop-overs of one day in Colorado Springs and special attention has been given to the schedule so that our party will pass through the scenic points of interest in daylight.

*Third train*, leave Chicago on August 25th, going via Burlington Route to Denver, thence via Denver & Rio Grande to Salt Lake City and Western Pacific to San Francisco as in route two. It will be noted that the two trains, that is, the trains leaving Chicago on the 24th and 25th, via Burlington Route, will meet in Colorado Springs and proceed from there as one or two trains according to the number who will take this route. It will also be noted that all the trains have been arranged so as to arrive in San Francisco one day prior to the opening of our convention.

There is a possibility that the number from the East will be sufficiently large to warrant the running of a special train through from New York, in which case the eastern and Chicago, and in vicinity parties will be consolidated and go as one train from Chicago. In the event that there is not a sufficient number to warrant the running of a special train through from New York, special sleepers will be provided for our use.

For the advance information of those interested in the trip, the Transportation Committees have endeavored to show briefly what the schedules of the trains will be. A circular outlining the trip in detail will be prepared some time in the near future and will be distributed generally to members of the Association.

To attend the Dental Congress and the Panama Exposition, it is understood that a reduction of fare is made for transportation to San Francisco from any point in United States and Canada.

There is no special train returning. It is therefore necessary to decide your return route when purchasing ticket on either of the following schedules.

TRAIN SCHEDULE I.

Via Boston & Albany.

Lv. Boston .................... 2:00 P. M., August 20th

Via New York Central, "Wolverine."

Lv. New York .................. 5:00 P. M., August 20th
Ar. Albany .................... 8:15 P. M., " "

(Connect with trains from Boston and other points in New England States.)

Via New York Central, "Wolverine."

Ar. Schenectady ............... 8:47 P. M., August 20th

Ar. Utica ..................... 10:23 P. M., " "

Ar. Syracuse .................. 11:40 P. M., " "

Ar. Rochester ................. 1:20 A. M., " 21st

Ar. Buffalo ................... 3:10 A. M., " "

(eastern time)

Ar. Detroit ................... 7:10 A. M., " "

(central time)

Ar. Chicago ................... 2:00 P. M., " "

(central station)

Via Chicago, Burlington & Quincy.

| | | | |
|---|---|---|---|
| Lv. Chicago | 6.10 P. M., | August | 21st |
| Ar. Kansas City | 8:00 A. M., | " | 22nd |

Via Atchison, Topeka & Santa Fe.

| | | | |
|---|---|---|---|
| Lv. Kansas City | 11:00 A. M., | August | 22nd |
| Ar. Colorado Springs | 6:30 A. M., | " | 23rd |
| Lv. Colorado Springs | 8:30 P. M., | " | 23rd |
| Ar. Albuquerque | 1:20 P. M., | " | 24th |
| Lv. Albuquerque | 2:00 P. M., | " | 24th |
| Ar. Isleta | 2:30 P. M., | " | 24th |
| Lv. Isleta | 4:00 P. M., | " | 24th |
| Ar. Grand Canyon | 5:00 A. M., | " | 25th |
| Lv. Grand Canyon | 8:00 P. M., | " | 25th |
| Ar. Redlands | 12:30 P. M., | " | 26th |
| Lv. Redlands | 2:30 P. M., | " | 26th |
| Ar. Riverside | 3:30 P. M., | " | 26th |
| Lv. Riverside | 11:59 P. M., | " | 26th |
| Ar. San Diego | 7:00 A. M., | " | 27th |
| Lv. San Diego | 11:59 P. M., | " | 27th |
| Ar. Los Angeles | 7:00 A. M., | " | 28th |

Via Southern Pacific.

| | | | |
|---|---|---|---|
| Lv. Los Angeles | 8:00 P. M., | August | 28th |
| Ar. San Francisco | 9:45 A. M., | " | 29th |

Railway fare from New York to San Francisco via the above route and returning via any direct route (not including Grand Canyon) .......... $98.80

The Wolverine—Fast Express—New York to Chicago, extra fare .......... 6.00

Those desiring a less expensive train to Chicago can leave Grand Central Terminal 2:00 P. M., August 20th, due Chicago 5:00 P. M., August 21st, with no extra fare.

Railway fare from Chicago to San Francisco, excluding Grand Canyon, going via the above route and returning via any direct route .......... $62.50

| | |
|---|---|
| Side trip from Williams to Grand Canyon and return | 7.50 |
| Lower berth from New York to Chicago | 5.00 |
| Lower berth, Chicago to San Diego (estimated) | 18.50 |
| Lower berth, San Diego to Los Angeles | 1.50 |
| Lower berth, Los Angeles to San Francisco | 2.50 |

Several fraternities and many members of the National Association are to travel by this route.

Special plans are being made by Dr. J. P. Marshall, 7401 Hazel Ave., Maplewood, St. Louis, Mo., for the accommodation of dentists from St. Louis and southern points to join these scheduled trains.

From Philadelphia and adjacent points on Pennsylvania R. R. connection can be made with these schedule trains going west from Chicago.

### Train Schedule II.

Via Chicago, Burlington & Quincy.

| | | | |
|---|---|---|---|
| Lv. Chicago | 11:00 P. M., | August | 24th |
| Ar. Denver | 7:00 A. M., | " | 26th |

Via Denver & Rio Grande.

| | | | |
|---|---|---|---|
| Lv. Denver | 8:00 A. M., | August | 26th |
| Ar. Colorado Springs | 10:30 A. M., | " | 26th |
| Lv. Colorado Springs | 10:30 A. M., | " | 27th |
| Ar. Salt Lake City | 12:30 P. M., | " | 28th |

Via Western Pacific.

| | | | |
|---|---|---|---|
| Lv. Salt Lake City | 1:00 P. M., | August | 28th |
| Ar. San Francisco | 5:00 P. M., | " | 29th |

| | |
|---|---|
| Railroad fare from Chicago to San Francisco going via the above route and returning via any direct route | $62.50 |
| Returning via Portland, Oregon | 80.00 |
| Lower berth, Chicago to San Francisco (estimated) | 15.00 |

TRAIN SCHEDULE III.

Via Chicago, Burlington & Quincy.

| | | | |
|---|---|---|---|
| Lv. Chicago | 11:00 P. M., | August | 25th |
| Ar. Denver | 7:00 A. M., | " | 27th |

Via Denver & Rio Grande.

| | | | |
|---|---|---|---|
| Lv. Denver | 8:00 A. M., | August | 27th |
| Lv. Colorado Springs | 10:30 A. M., | " | 27th |
| Ar. Salt Lake City | 12:30 P. M., | " | 28th |

Via Western Pacific.

| | | | |
|---|---|---|---|
| Lv. Salt Lake City | 1:00 P. M., | August | 28th |
| Ar. San Francisco | 5:00 P. M., | " | 29th |

Rates will be the same as Route II, except that a lower berth from Chicago to San Francisco will be $13.00.

Those desiring to go by the Northern Routes via Portland, returning by a central or southern route, there is an added charge of $17.50.

Applications for space starting from Boston and vicinity should be addresed to Mr. C. E. Colony, City Ticket Agent, B. & A. Road, Boston, Mass.

Or from New York, Mr. W. V. Lifsey, General Eastern Passenger Agent, New York Central Lines, 1216 Broadway, New York.

Starting from Chicago, address Mr. A. J. Puhl, General Passenger Agent, C. B. & Q., 141 South Clark St., Chicago, Illinois.

Starting from Kansas City or southwestern points joining at Kansas City, address Mr. G. W. Hagenbuch, General Agent, Santa Fe Route, 905 Main St., Kansas City, Mo.

Starting from New York or New Orleans to San Francisco by the Southern Pacific Company Routes.

Ships of the Southern Pacific Steamship Line leave New York on Wednesdays and Saturdays at twelve noon for New Orleans arriving the following Monday and Thursday respectively and from New Orleans continue on the Southern Pacific R. R. to San Francisco.

The rate by this route from New York to San Francisco, returning by any central or southern route is ........................................$94.30
Or according to the route and train selected from Chicago east .................................... 96.55

This rate includes berth and meals on steamer from New York to New Orleans.

There is an added Pullman charge for lower berth from New Orleans to San Francisco of $11.50.

For space on steamer, address Mr. L. H. Nutting, General Passenger Agent, Southern Pacific Co., 1158 Broadway, New York.

Starting from New Orleans, address General Passenger Agent of Southern Pacific Co., Mr. J. H. R. Parsons, Camp and Poydras Sts., New Orleans, La.

The Transportation Committees would suggest that before purchasing tickets that each person verify the particular schedule of the route chosen in going and in returning.

Transportation Committee, National Dental Association:

Dr. Victor H. Jackson, *Chairman*, New York.
Dr. H. F. Hoffman, Denver, Colo.
Dr. Jos. D. Eby, Atlanta, Ga.
Dr. D. C. Bacon, Chicago, Ill.
Dr. Henry W. Weirick, San Francisco, Cal.
Dr. J. P. Marshall, St. Louis, Mo.

Transportation Committee, Panama-Pacific Dental Cong.:

Dr. Henry W. Weirick, *Chairman*, San Francisco.
Dr. Harry P. Evans, New York.
Dr. Alpheus R. Brown, Boston, Mass.
Dr. E. M. Carson, St. Louis, Mo.
Dr. F. W. Gethro, Chicago, Ill.
Dr. Jos. D. Eby, Atlanta, Ga.
Dr. Chas. F. Fiset, Seattle, Wash.
Dr. R. W. Berthel, St. Paul, Minn.

